# Cannabis, One Health, and Veterinary Medicine: Cannabinoids’ Role in Public Health, Food Safety, and Translational Medicine

**DOI:** 10.5041/RMMJ.10388

**Published:** 2020-01-30

**Authors:** Sivan Ritter, Lilach Zadik-Weiss, Osnat Almogi-Hazan, Reuven Or

**Affiliations:** 1 Independent Consultant, Tel Aviv, Israel; 2 Independent Consultant, Amsterdam, The Netherlands; 3 Laboratory of Immunotherapy and Bone Marrow Transplantation, Hadassah Hebrew University Medical Center, Jerusalem, Israel

**Keywords:** Animal models, cannabis, One Health, public health, translational medicine, veterinary medicine

## Abstract

Public health is connected to cannabis with regard to food, animal feed (feed), and pharmaceuticals. Therefore, the use of phytocannabinoids should be examined from a One Health perspective. Current knowledge on medical cannabis treatment (MCT) does not address sufficiently diseases which are of epidemiological and of zoonotic concern. The use of cannabinoids in veterinary medicine is illegal in most countries, mostly due to lack of evidence-based medicine. To answer the growing need of scientific evidence-based applicable medicine in both human and veterinary medicine, a new approach for the investigation of the therapeutic potential of cannabinoids must be adopted. A model that offers direct study of a specific disease in human and veterinary patients may facilitate development of novel therapies. Therefore, we urge the regulatory authorities—the ministries of health and agriculture (in Israel and worldwide)—to publish guidelines for veterinary use due to its importance to public health, as well as to promote One Health-related preclinical translational medicine studies for the general public health.

## INTRODUCTION

The term animal/murine model is commonly used in medical studies. The scientific paradigm of preclinical trials has not changed throughout the years. Preclinical trials using rodent models are limited in their ability to predict outcomes in human patients. Many medications developed based on rodent models fail to demonstrate clinical efficacy in humans; hence these models are neither time- nor cost-effective. There are numerous reasons why laboratory animal models fail to model human reactions to drugs properly. As opposed to in humans and pets, disease does not occur naturally in animal models, and therefore the disease is not the same in the test subjects as in humans. Environmental risk factors for disease occurrence (and environmental factors influencing patients at home) are not comparable between animal models in a lab and human patients at home or in a hospital.

The One Health Initiative recognizes that both human and animal health are connected, together with the ecosystem.^1^ From this perspective, a better, “One Health” model for drug testing might be found in animals with naturally occurring diseases, treated as a part of their life routine, in the same environment as humans, allowing for the simultaneous development of drugs for human and animal patients, thereby reducing time to development of drugs for humans, reducing associated costs, *and* contributing to animal welfare.^2^

## ONE HEALTH, PUBLIC HEALTH, AND FOOD SAFETY

The One Health Initiative mission statement emphasizes the recognition that human health, animal health, and ecosystem health are inextricably linked, and seeks to enhance cooperation and collaboration between professionals of all related fields.^2^ Few publications deal with the zoonotic and epidemiological aspects of public health regarding medical cannabis therapy (MCT). Public health in general—and food safety specifically—demonstrates the close relationship between veterinary and human medicine. In the fields of clinical, occupational, and environmental medicine there are also rising concerns regarding hazards of cannabis production in which there is an exposure to several contaminants including microbes, heavy metals, and pesticides.^3^–^5^ A recent example of the important role of public health with regard to cannabis can be found in the Denver Department of Public Health & Environment investigation, which found potentially unsafe levels of yeast and mold in samples of dried marijuana. This led the Denver Department of Public Health & Environment to issue and oversee a recall process to remove potentially contaminated products from commercial circulation; to date the location list of retailers to which contaminated material was distributed includes 144 retail stores, 3 cultivation facilities, and 11 manufacturing facilities.^6^

## THE LEGAL PROBLEM OF VETERINARY MEDICAL CANNABIS IN ISRAEL

The use of cannabinoids in veterinary medicine in Israel is illegal. Special guidelines published by the Israeli Ministry of Health set the terms for human use. There are two possible approaches that could be used to legalize cannabis for veterinary patients. The first approach would be to publish guidelines for veterinary use by the ministries of health and agriculture. These guidelines would define the following: (a) Who is allowed to prescribe cannabinoids to animals in Israel—all veterinarians versus only veterinarians authorized to prescribe cannabinoids? (b) Who will sell them—veterinary clinics versus pharmacies? (c) How will cannabinoid use be regulated to avoid abuse by owners of the animals? (d) Which animals can receive cannabinoids (addressing the issue of food-producing animals)? and (e) What is the procedure necessary to approve each veterinary cannabis prescription? A second approach would be to amend the criminal law and legalize cannabinoids, or at least cannabidiol (CBD) without restrictions. Both paths would enable veterinary patients to receive MCT, with the choice dependent on the general approach and policy of the regulator regarding cannabinoid use in Israel.

## CURRENT FOOD SAFETY CONCERNS AND THEIR INTERFACES WITH PUBLIC HEALTH

Chemicals that are present in foodstuffs can be intentionally added (i.e. food additives, or the illegal addition for adulterant purposes), are present as residues from defined uses (e.g. pesticides and veterinary drugs) or are contaminants (formed during production, processing, storage or stemming from the environment). Governments operating a food safety programme do so to ensure that food available to the population is safe and compliant with established standards.^7^

The term “from farm to fork” describes the importance of food safety along all stages of food production. We suggest that food safety should also address the connection between animal feed (feed), food, and the health of veterinary and human patients including the gut flora of patients—”from feed to farm to fork to flora” (FFFF). When dealing with food or feed the main concerns relate to safety and quality. Such an example can be found in the International Agency for Research on Cancer 2018 monographs, in which positive associations were observed between consumption of red meat to colorectal, pancreatic, and prostate cancers; there was also sufficient evidence to indicate that processed meat caused colorectal cancer, and a positive association was found to stomach cancer.^8^ These conclusions demonstrate the importance of the FFFF impact, with further studies required in order to prevent or reduce the incidence of diseases affecting morbidity and mortality rates within the general population—all of which could be translated into costs to public health due to an increasing disease burden.^9^–^12^

## EDIBLE CANNABINOID PRODUCTS

Some cannabinoid products are consumed *per os*, which causes disagreement on their right definition; however, there is no controversy over the fact they should be legally defined and regulated.^13^–^15^ Cannabis, as it relates to public health, clearly impacts food, feed, and pharmaceuticals. Therefore, the use of phytocannabinoids should be examined from a One Health perspective. It is hard to ignore the availability of cannabis-infused foods and drinks (i.e. “edibles”). Edibles lack a legal distinct definition, yet countless products are available on the market for both humans and animals.

## THE LEGAL DEFINITION OF SUPPLEMENTS

Feed and food supplements are concentrated sources of nutrients or other substances with a nutritional or physiological effect. Supplements are intended to correct nutritional deficiencies, to maintain an adequate intake of certain nutrients, and to support specific physiological functions. Supplements are not medicinal products and as such cannot claim to exert a pharmacological, immunological, or metabolic action. Their use is not intended to treat or prevent diseases in humans or to modify physiological functions. Supplements are marketed in “dose” form (i.e. pills, tablets, capsules, liquids in measured doses). They may include vitamins, minerals, amino acids, essential fatty acids, fiber, and various plants and herbal extracts.^14^,^16^ “The global dietary supplement market is projected to reach 194 billion USD by 2025,”^17^ and such growth projection emphasizes the need to fully understand the implications and assess possible risks to public health that might arise in a certain population.

## SUPPLEMENTS AND CLINICAL ENDOCANNABINOID DEFICIENCY

Since supplements are intended to correct nutritional deficiencies, the theory of clinical endocannabinoid deficiency (CED) supports registering cannabinoids as supplements. According to Russo,^18^–^20^ humans have an underlying endocannabinoids (eCB) tone reflecting levels of N-arachidonoylethanolamine (AEA or anandamide) and of 2-arachidonoylglycerol more familiarly known as 2-AG—their production, metabolism, abundance, receptor status, etc. Under certain conditions, congenital or acquired, eCB tone becomes deficient, producing pathophysiological syndromes. Comparable deficiency in eCB levels might be manifested similarly in certain disorders that display predictable clinical features as sequelae of this deficiency. The greatest evidence for CED is seen by its presence in migraine (AEA levels in the cerebrospinal fluid), fibromyalgia (central eCB hypofunction in the spinal cord), and irritable bowel syndrome (IBS; CB1 gene, CNR1 gene mutations). Other CED-related conditions include posttraumatic stress disorder, Parkinson’s disease, diabetes, multiple sclerosis, and Huntington’s disease. Russo has suggested that eCB may play a role in neurotransmitter deficiencies, including the acetylcholine deficiency found in Alzheimer’s disease, and the serotonin and norepinephrine deficiencies found in depression.^18^,^19^ In such cases, therapy options may include fatty acid amide hydrolase (commonly referred to as FAAH) inhibitors (that will raise AEA levels), exogenous cannabinoids, and integral lifestyle approaches (such as low-impact aerobic exercise regimens, dietary manipulations with probiotics, prebiotics, etc.).^19^

## ANIMAL FEED AND CANNABIS

As part of the concern regarding chemical residues in food, a ban of antibiotic growth promoters in animal feed was implemented in EU countries in 2006. Essential oils (EOs) are an important alternative to antibiotics in animal diets, and are being further researched and used in livestock, swine, and poultry feed, as well as in aquaculture (fish farming).^21^–^26^ In veterinary medicine, the term phytogenic feed additives encompasses combined bioactive ingredients and flavoring substances (i.e. EOs, spices, herbs, or plant extracts) which influence the gut flora—for performance and yield enhancements (reducing oxidative stress, improving feed conversion rate, as well as improving digestibility, growth rate, and quality of derived products such as meat, eggs, dairy, and fish products).^12^ In food-producing animals, whose yield is consumed by humans on a large scale, hemp-derived feed (seed, seed cake/meal, seed oil, whole plant) was included in the feed prior to its de-legalization and is now being carefully reintroduced, under certain technical process, into poultry feeding regimes as well as for pigs, ruminants, and fish.^27^–^33^ According to the European Food Safety Authority (EFSA), varieties allowed for hemp cultivation in Europe must not exceed 0.2% delta-9-tetrahydrocannabinol (THC),^25^ and according to the US Food and Drug Administration (FDA) hemp THC concentration should not exceed 0.3% on a dry weight basis.^26^

Regarding animal (including pet) feed, the FDA is aware of some cannabis products being marketed as animal health products. We want to stress that FDA has not approved cannabis for any use in animals, and the agency cannot ensure the safety or effectiveness of these products.^34^

At this time, there are no approved food additive petitions or ingredient definitions listed … for any substances derived from hemp, and we are unaware of any GRAS [generally recognized as safe] conclusions regarding the use of any substances derived from hemp in animal food.^35^

The American Veterinary Medical Association report of January 2018 stated that the FDA had issued warning letters to companies selling products for animals containing CBD; many products did not contain the levels of CBD they claimed, with some containing 0.0025% CBD while others contained 25%–35% CBD.^36^

## NUTRACEUTICALS

Nutraceuticals were first defined by DeFelice as “Food, or parts of a food, that provide medical or health benefits, including the prevention and treatment of disease,”^37^ thus having a pharmacological beneficial effect on health. Nutraceuticals are not a legally defined category like *food supplements* (such as herbal products, pre- and probiotics, functional foods, etc.). In order to evaluate and regulate their safety and efficacy, nutraceuticals must be legally defined. There is a risk of “over-interpretation” of claimed health benefits of supplements, which may not be properly substantiated by safety and efficacy *in vivo* data as required for pharmaceuticals.^38^,^39^ Despite the lack of a legally accepted definition, there is a growing number of publications regarding clinical research fields and the pharmacological beneficial effects of nutraceuticals on health—for example, bone health,^40^ vascular health,^41^,^42^ immunity,^22^,^23^,^43^,^44^ applied physiology,^45^ dermatology,^46^ and in veterinary medicine.^44^,^47^,^48^ The lack of legal recognition for the term nutraceuticals has led to nutraceutical products, including cannabis, being registered differently, depending on specific state legislation, either as a pharmaceutical drug, or as a food/feed supplement/additive. For example: “Based on available evidence, FDA has concluded that THC and CBD products are excluded from the dietary supplement definition”^49^ and “… it is a prohibited act to introduce or deliver for introduction into interstate commerce any food (including any animal food or feed) to which THC or CBD has been added.”^50^ Furthermore, in November 2019 the FDA issued warning letters to 15 companies for illegally selling various products containing cannabidiol; the letter detailed safety concerns. Violations included marketing unapproved new human and animal drugs, selling CBD products as dietary supplements, and adding CBD to human and animal foods.^51^–^53^

## CANNABIS MEDICINAL PROFILE AND PHYTOCONSTITUENTS

Cannabis medicinal profile phytoconstituents include phytocannabinoids, which are plant-derived (botanical origin) chemical compounds which interact with the (eCB) system.^47^ The “entourage effect” is the total sum of all phytochemical interactions of all the phytoconstituents, differentiating from the bioactivity and effect of each component alone. Other bioactive constituents include phenolic compounds, flavonoids, and terpenes which have potential free radical scavenger activity and are omnipresent groups of plant secondary metabolites.^54^,^55^ Flavonoids (also found in fruits, vegetables, grains, bark, roots, stems, flowers, tea, and wine) have been shown to have diverse medicinal effects and act synergistically with terpenoids.^56^–^61^ Cannflavin A, B, and C are methylated isoprenoid flavones unique to cannabis. A recent study demonstrated a concentration-dependent hormetic and neuroprotective role of cannflavin-A against amyloid-β-mediated neurotoxicity, associated with an inhibition of amyloid-β fibrillization, suggesting a line of research in regard to Alzheimer’s disease, and noted that the geranylated flavonoids generally displayed a comparatively potent neurotoxicity not observed with many conventional flavonoids *in vitro*.^62^ Terpenoids are a major component of EOs, lipophilic, and can cross the blood–brain barrier. Terpenes exert diverse medicinal effects.^55^,^63^–^75^ Terpenoids are also flavor and fragrance components common to human and pet diets and are considered by the US FDA to be generally recognized as safe (GRAS). The use of terpenoids in food-producing animal feed is being researched due to bio-technological effects on food quality and safety as well as for performance enhancement such as weight gain; feed conversion rate; nutrient absorption; gut-lining morphology; relief of intestinal immune defense in gut-associated lymphoid tissue due to antibacterial, antiviral, antifungal properties; intestinal microbiota health; meat lipid oxidation; milk composition; malodors and off-flavor in aquaculture; and palatability of feed.^76^–^79^

## CANNABIS, ONE HEALTH, AND FOOD SAFETY ASPECTS

From a One Health perspective there are questions which require further investigation: Is the current food safety regulation sufficient and satisfactory in ensuring public health, given the current low use of over-the-counter bioactive molecules? Do relative costs (also for the end-user) versus public health concerns justify registering all products containing bioactive molecules as pharmaceuticals rather than as supplements/additives? Should products containing bioactive molecules above a certain limit of total ingredients be regulated separately, including cannabinoids? How should the lack of sufficient comparative science to determine the long-term effects of nutraceuticals in both human and veterinary patients be addressed? What is the advised nutrition for immunocompromised populations when considering CED (what requires base-line establishment prior to treatment)? What is the recommended food composition profile? Are there necessary supplements, or supplements that should be avoided, with concurrent treatments (i.e. citicoline^80^)? Which medical considerations should be included in treatment considerations (malabsorption, maldigestion, anorexia, inflammatory bowel diseases [IBDs], etc.)? What nutritional and food-safety advice should be given along with the cannabis prescription? What are the environmental risks (risks of exposure to contaminants) that patients might share in their disease etiology? Can veterinary patients with a similar natural occurring disease shed more light on the pathology? These questions demonstrate some of the FFFF effect which needs to be considered. We urge the regulatory authorities and the policymakers to address these questions and provide suitable guidelines for cannabis-based products in view of all aspects of public health.

## FOOD SAFETY REQUIREMENTS

During the 1990s, major food safety adverse events (such as the bovine spongiform encephalopathy,^81^ dioxin,^82^ and Listeria^83^ outbreaks) led to establishment of the Global Food Safety Initiative.^84^ The Food Safety System Certification FSSC22000,^85^ which is a set of controls to assure food processing safety and sanitation, is now an obligatory requirement: the FDA Food Safety Modernization Act (FSMA)^86^ has adopted the Global Food Safety Initiative’s certification, resulting in significant international trade implications on the global food supply chain—from the perspective of foreign supplier verification programs—as well as on the regulatory requirements of different markets for Hazard Analysis and Risk-based Preventive Controls programs for both human food and animal feed.^87^,^88^

The risks of food or feed contamination are present from farm to fork. They can result from environmental contamination (pollution of water, soil, or air) and require prevention and control throughout the food chain: at the farm level (contaminated animal feed, parasites that infect food-producing animals, milk contaminated through contact with feces or environmental dust, animal skin and fur contaminated by feces and the environment), during slaughter (intestinal contents and fecal contamination), during processing (cross-contamination, infected employees), during preparation (improper use of utensils or working surfaces), and in storage and during distribution (cross-contamination). The risk of zoonoses (i.e. listeriosis, campylobacteriosis, salmonellosis as well as *E. coli*, parasitical, viral, and prion infections) via contaminated foodstuffs or through contact with infected animals or their secretions is considered to be high in products of animal origin.^89^

## NOVEL PUBLIC HEALTH AND EPIDEMIOLOGY APPLICATIONS FOR CANNABINOIDS

Clinical indications in human patients for cannabis in Israel include oncology, gastroenterology, neuropathic pain, infectious diseases (human immunodeficiency virus [HIV]/acquired immunodeficiency syndrome [AIDS]), neurology, palliative care, and psychiatry. Interestingly, these correlate to risk groups for foodborne illness (which include patients suffering from cancer, diabetes, HIV, and other immunocompromised populations).^90^ According to the World Health Organization (WHO),

Unsafe food containing harmful bacteria, viruses, parasites or chemical substances, causes more than 200 diseases—ranging from diarrhoea to cancers. An estimated 600 million—almost 1 in 10 people in the world—fall ill after eating contaminated food and 420,000 die every year, resulting in the loss of 33 million healthy life years (DALYs). Diarrhoeal diseases are the most common illnesses … causing 550 million people to fall ill and 230,000 deaths every year.^91^

The most common clinical presentations are gastrointestinal symptoms; however, such diseases can also involve neurological, gynecological, immunological and other symptoms. According to the EFSA, “between one third and one half of all human infectious diseases have a zoonotic origin … about 75% of the new diseases that have affected humans … have originated from animals or products of animal origin.”^92^ Cannabinoids may potentially address such diseases and vice versa—epidemiological data can assist in identifying needs for further MCT research for the benefit of public health.

### Listeriosis

Listeriosis severely affects pregnant females, new-borns, the elderly, and immunocompromised individuals. It is widely distributed in nature; *Listeria spp*. can be found in soil, water, vegetation, and the feces of some animals, and it can contaminate foods.^93^–^95^ Listeriosis was the cause of death in several outbreaks (i.e. contaminated soft cheese sickened 142 people, killed 10 new-borns and 18 adults, and caused 20 miscarriages in one outbreak; in another outbreak, contaminated cantaloupes caused 33 deaths^96^). Since Listeria can thrive in cold temperatures, high-risk foods include: deli meats and ready-to-eat meat products (such as cooked, cured, and/or fermented meats and sausages), soft cheeses, and cold smoked fishery products.^95^,^96^ Additionally, *Cannabis sativa L.* EOs were found to demonstrate, both *in vitro* and *in vivo*, some attenuation of *Listeria monocytogenes* virulence,^97^ which could potentially be a novel strategy to reduce antimicrobial resistance in general, as well as reducing biological contaminants in food-producing establishments.

### Chagas Disease

Chagas disease/American trypanosomiasis (vector-borne disease, which is transmitted by the parasite *Trypanosoma cruzi*), is a zoonosis of high interest for public health and for veterinary medicine (as dogs are frequently infected with *T. cruzi*); prevalence of *T. cruzi* infection is associated with increased risk of Chagas disease in humans.^86^,^88^

According to the WHO,

about 6 million to 7 million people ... worldwide are estimated to be infected with *...* the parasite that causes Chagas disease … Up to 30% of chronically infected people develop cardiac alterations and up to 10% develop digestive, neurological or mixed alterations ... Blood screening is vital to prevent infection through transfusion and organ transplantation.^98^

Cannabinoid compounds have been shown to inhibit parasite proliferation, growth, and invasion.^99^ *Trypanosoma cruzi* invades cardiac cells via calcium-dependent G protein-coupled pathways and may lead to carditis, which may be of autoimmune origin: “cannabinoids can block cardiac cell puncture repair mechanisms, thereby inhibiting trypanosome invasion as predicted by the mode of drug action, but also inhibit immune cell effector functions, offsetting the benefit of inhibition parasite cell invasion.”^100^ The Pan American Health Organization Panama country report stated that the highest *morbidity* was due to hypertension and that new cancers was third highest; the leading causes of *mortality* included malignant neoplasms, followed by ischemic heart diseases and by cerebrovascular diseases.^101^,^102^ However, it is not yet known if there is a correlation between possible outcomes of chronic Chagas infections and cardiovascular morbidity and mortality rates. Chagas disease in dogs is also of high prevalence in Latin America, and cardiac alterations are often present.^103^–^106^ This suggests that a translational medicine study of both veterinary patients and disease progress in a natural occurring disease, and of human patients, may provide a better understanding of the disease and contribute to the development of novel treatments.

### Leishmaniasis and Malaria

Translational studies of leishmaniasis in both human and veterinary patients offer great potential, since the condition is endemic in 88 countries (including Europe^107^ and Israel^108^–^111^) and on four continents: “an estimated 12 million cases of leishmaniasis exist worldwide with an estimated number of 1.5–2 million new cases occurring annually; 1–1.5 million cases of cutaneous leishmaniasis and 500,000 cases of visceral leishmaniasis.”^112^ Leishmania is becoming more urban and peri-urban and is not limited to rural areas. In Europe it is also important to note the impact caused by the influx of refugees mostly from Syria; the high infection rates noticed in refugee camps indicate an increased risk of infection among the local population.^112^–^121^ In addition, the number of cases of Leishmania/HIV co-infection has increased in recent years.^112^ Interestingly, *Cannabis sativa* plants were consumed by blood-sucking phlebotomine sand flies, the primary vectors of Leishmania, much more frequently than expected (i.e. since both sexes of flies consume plant-derived sugar meals, it is probable that *C. sativa* is highly attractive to sand flies).^122^ Some studies suggest that the EOs (especially terpenoids) are the primary phytoconstituents responsible for arthropod deterrence.^123^ Nevertheless, other polyphenolic compounds were found to have an anti-leishmanial effect^124^: THC was found to exert some nominal deterrence; as it is toxic to insects,^123^ it might be a possible alternative to current insecticides, pesticides, and repellants. However, there are few studies on this topic. Nevertheless, it was noted that 5-acetyl-4-hydroxycannabigerol displayed strong anti-leishmanial activity,^125^ and that 1′S-hydroxycannabinol activity was found to have a moderate anti-methicillin-resistant *Staphylococcus aureus* effect, a moderate anti-leishmanial effect, and mild anti-malarial activity against *Plasmodium falciparum*, which transmits malaria.^126^ Malaria is another zoonosis with great research potential as very few cannabis studies couple public health with veterinary medicine (in 2017 alone, there were 219 million cases and 435,000 deaths, with allocated resources of US$3.1 billion).^127^–^131^

### Multiple Myeloma

With regard to multiple myeloma, the epidemiological data trends in Latin America are most interesting. Increasing mortality has been verified in seven countries. Identified risk factors included age over 60 years old, male sex, and ethnicity, as well as occupational and environmental exposure to benzene, pesticides, dichlorodiphenyltrichloroethane, commonly known as DDT, and petroleum derivatives.^132^ A case-control study conducted in Uruguay demonstrated that red meat, salted meat, and milk were positively associated with risk of lymphoid cancers, and that plant foods, particularly total fruits, and alcoholic beverages were protective, thereby concluding that these foods could play a significant role in the etiology of lymphoid malignancies, as part of the FFFF effect.^133^ Designated cannabinoid formulations for treatment of multiple myeloma are currently being patented.^134^–^137^ Among other possible multiple myeloma risk factors were overweight and obesity, low fish and green vegetable consumption, AIDS, and herpes zoster infection,^132^ which relate conceptually to CED. Different fatty acids react differently to cytotoxic effects of drugs; therefore, there is a need to consider the interactions of different fatty acids—whether as food or as supplements (which potentially may be aimed to correct CED)—with concurrent treatments in two aspects: (a) regarding the effects of different fatty acids on cytotoxic effects of drugs; and (b) regarding the negative effects of drugs on antitumor actions of specific fatty acids.^138^

Furthermore, a recent study found that cannabinoid-based treatments suppress rather than provoke lymphocyte proliferation; influence cytokine secretion; and that pure cannabinoids exhibit a superior effect *in vitro*, but that in a syngeneic transplantation model THC-high and CBD-high cannabis extracts treatment reduced the severity of graft versus host disease, and improved survival significantly better than pure cannabinoids.^139^

Multiple myeloma is an uncommon lymphoproliferative disease reported in several species including dogs, cats, and horses.^140^–^143^ It differs between species and is considered a heterogeneous disease with a different prognosis, clinical course, and response to therapy both within and between species.^141^ Veterinary patients diagnosed with multiple myeloma could potentially help overcome the lack of success in translating promising results in rodents and in other laboratory species.

### Neurogenerative and Geriatric Diseases

The increased prevalence of chronic diseases, especially neurogenerative and geriatric diseases, is attributed to the increased life expectancy of both humans and of pets. Geriatric conditions such as cognitive dysfunction syndrome, age-associated decline in renal function, and impairment of cell-mediated immune function have been studied in veterinary patients (including the additional challenge of under-diagnoses of geriatric pets as being truly geriatric).^144^–^151^ Pets have shorter lifespans and experience a more rapid progression of diseases, making the study of natural occurring diseases in veterinary patients relatively expeditious compared to in humans, as well as more cost-effective.

## VETERINARY MEDICINE AND MEDICAL CANNABIS THERAPY

Veterinary medicine is lagging behind human medicine with regard to MCT due to the lack of sufficient evidence-based studies. Very few studies have been published, and most of them relate to CBD.^152^–^156^ Nevertheless, there are many anecdotal case studies about successful cannabinoid treatment in pets for similar indications in humans. Additionally, some veterinary physicians are biased against MCT, which could be associated with the lack of approved MCT training for professionals and students.^157^ In parallel to a growing demand from pet owners for cannabis—in part due to the industry-driven market—there is an expanding availability of pet-edible cannabinoid-based products. As mentioned above, Israeli legislation prohibits veterinarians from prescribing, dispensing, or recommending cannabinoid-based products to animals. The market projection for CBD pet products in the US alone has been noted to be 7% out of a forecast of US$24 billion annual sales,^158^ indicating a need for veterinary training since animals are already receiving these products from their owners without proper medical supervision. A significant correlation was found between the number of medical marijuana licenses and marijuana toxicosis in pets (via ingestion of edibles [~66%], of plant material [~19%], and of medical cannabis preparations and/or prescription medications such as dronabinol and nabilone [~9%]^159^). Ingestion of THC-containing edibles has resulted in two deaths.^160^ However, the reported cases had become further complicated, and the exact cause of death was not determined; hence it was concluded that no deaths associated with marijuana had been reported.^159^ It is worth noting that children often share the same risks as household pets, including exposure to toxicants.

### The Endocannabinoid System in Animals

Numerous scientific publications exist regarding the endocannabinoid system (ECS) in various laboratory animal species; these publications support safety and therapeutic potential—which could be actually translated to any vertebrate species, thus representing the idea of translational medicine in regard to the ECS. The ECS has been described in invertebrates,^161^ including molluscs,^162^ protozoa,^163^,^164^ nematodes, onychophorans, crustaceans,^165^ and sea-urchins,^166^ and some species of cnidarians^165^,^168^; in lower vertebrates,^161^ including amphibia (salamanders, frogs) and other aquatic organisms^161^ (such as goldfish,^167^ zebra-fish,^169^); and in poultry^161^ (pigeons,^170^ chickens, zebra-finch). Salzet and colleagues noted that the “Cannabinoid system in all probability originated in ‘simple’ and primitive animals to control physio pathological responses, either similar or entirely different from those that are modulated by endocannabinoids in higher vertebrates and mammals”^161^ and that “Anandamide, by acting at CB1-like receptors, seems to induce often similar responses in both mammalian and invertebrate cells, particularly for what concerns the modulation of *neurotransmitter release* and action and the *control of immune cell function* [emphasis added].”^171^ Differences of cannabinoid receptors’ density and distribution in animals^172^–^174^ can account for therapeutic effects in animal models that do not translate well into human models.

It is important to note species differences, such as the case regarding the recently identified human GABAergic interneuron subtypes called “rosehip cells,” which present an immunohistochemical profile not seen in rodents, and which, despite previous studies on cortical interneurons, showed functional presynaptic expression of CB1r in rodents; application of CB1r antagonist was ineffective in modulating rosehip cell-evoked inhibitory postsynaptic potentials.^175^ Interspecies differences in eCB signaling were also found, suggesting a species profile of eCB signaling from the highest to lowest: *rat > chicken > mouse > [human = dog]*. The highest signaling was noted in the rat, in which cannabis extracts induced reliable epileptiform convulsions and caused clear signaling down-regulation. The lowest signaling was noted in the dog, in which seizures did not occur (ataxia, tremors, hypoactivity, and other central nervous system signs were observed^176^).^177^

Cannabis intoxication in pets most commonly affects dogs (96%) and is uncommon in cats (3%).^178^ The minimum lethal oral THC dose for dogs is more than 3 g/kg.^176^,^179^ The median lethal dose, commonly known as LD50, has not been established in dogs or cats.^179^ Onset of clinical signs is within minutes (via inhalation) and about 60 minutes (*per os*). The biological half-life is 30 hours, and excretion is within 5–15 days (85% in feces and 15% in urine); recovery post ingestion is within 24 hours in most cases but can be up to 72 hours.^159^,^176^,^179^

### Veterinary Medicine, Translational Medicine, and Medical Cannabis Treatment

The goal of translational medicine according to the European Society for Translational Medicine is:

… to combine disciplines, resources, expertise, and techniques within its three main pillars which are bench-to-bedside, bedside-to-bench and community; in order to promote enhancements in prevention, diagnosis, and therapies.^180^,^181^

Cannabinoids can be used in human medicine to treat many medical conditions, such as for anxiety disorders, as antiemetics, for modulation of blood pressure and heart rate, and in asthma, inflammation, cancer, and diabetes. However, we have not found published research regarding those uses in veterinary patients. Due to legal issues, cannabinoids cannot be used in veterinary medicine in Israel. In some countries MCT is available for veterinary patients, and there is some research demonstrating its beneficial effect.

Cannabinoids have been shown to ameliorate pain associated with osteoarthritis in dogs.^153^ Canine osteoarthritis is associated with hip dysplasia, as well as with low back pain. It is generally common in large-breed dogs, with a predisposition in certain breeds which display a higher incidence.^182^–^186^ In humans, treatment of low back pain with MCT has been found to be advantageous as compared to standardized analgesic therapy.^187^ These similarities warrant further dual veterinary–human research.^188^ Cannabidiol was also evaluated for seizure reduction in epilepsy in dogs, although the results were preliminary and require further research.^146^,^176^ Neonatology studies in piglets have demonstrated the effects of CBD after induced acute hypoxic ischemia for lung injury, for hypothermia, and in short-term brain damage, as well as for high-dose CBD post-exposure hypotension.^189^–^191^ While THC can be toxic to dogs and should be given cautiously, CBD was shown to be safe even at high doses.^148^,^150^

Autoimmune diseases and conditions involving chronic inflammation are challenging to treat in both humans and animals. The CB2 receptors are expressed in the immune cells of both humans and animals, therefore cannabinoids have been explored as a potential treatment for these conditions.^139^,^192^ Cannabinoid therapy has provided few improvements for these conditions, although further clinical research is needed. There is also growing interest in the potential of cannabinoids to treat HIV patients; investigation of feline immunodeficiency virus in cats could contribute to anti-retroviral therapy development.^193^–^197^ Another disease that shares similarities in veterinary and human patients is IBD. The clinical symptoms and molecular features have been found to be common in both animals and humans^198^–^203^; however, some animal breeds are more predisposed to IBD.^199^,^201^,^204^–^208^ There is speculation that IBD evolved partially due to an inappropriate immune response to bacterial and/or dietary antigens. Food sensitivities and allergies contribute to compromised immune function.^198^,^202^,^209^–^214^ Traditional treatments often exacerbate the disorder, thus patients have increased susceptibility to gastro-intestinal lymphoma.^194^,^215^–^221^ Cannabinoids exert multiple effects by modulating neurotransmitters and the immune response,^139^,^222^ and are a promising immunosuppressive and anti-fibrotic agent in the treatment of autoimmune disorders—and possibly IBD.

## SUMMARY

The similarities between many animal and human diseases justify translational studies that will benefit all species. Such studies have the potential for better and more cost-effective medication development. There are many diseases common to several species that can potentially be treated with cannabis, which represent a good starting point for translational studies. It is therefore imperative that legislation is passed to enable treating animals with cannabis.

## Abbreviations

AEA: N-arachidonoylethanolamine, anandamide
AIDS: acquired immunodeficiency syndrome
CBD: cannabidiol
CED: clinical endocannabinoid deficiency
eCB(s): endocannabinoid(s)
ECS: endocannabinoid system
EFSA: European Food Safety Authority
EOs: essential oils
feed: animal feed
FDA: US Food and Drug Administration
FFFF: from feed to farm to fork to flora
FSMA: FDA Food Safety Modernization Act
GRAS: generally recognized as safe
HIV: human immunodeficiency virus
IBD(s): inflammatory bowel disease(s)
MCT: medical cannabis treatment
THC: delta-9-tetrahydrocannabinol
WHO: World Health Organization.
